# Supplementary material for: MARK2/Par1b kinase present at centrosomes and retraction fibres corrects spindle off-centring induced by actin disassembly
Source: Open Biol. 2019 Jun 26;9(6):180263. doi: 10.1098/rsob.180263 (PMC6597755; doi:10.1098/rsob.180263)

Supplementary Movies

Movie S1: MARK2 is enriched at mobile membrane subdomains in interphase

Time-lapse TIRF (Total Internal Reflection Fluorescence) microscopy movie of a HeLa FRT-TO cell expressing MARK2-YFP WT protein. TIRF microscopy images taken once every second show MARK2-YFP's mobility as membrane patches through time. Time lapse images shown (with time-frames indicated) correspond to the cell in figure 1*d* (Time-frame; 0 sec) and figure S1.

Movies S2 & S3: Re-centering of spindles in Latrunculin-treated cells needs MARK2

Time-lapse deconvolution movies of HeLa (His-GFP; mCherry-Tubulin) cells treated with Control siRNA (Sup. Mov. 2) or MARK2 siRNA (Sup. Mov. 3) and exposed to low doses of Latrunculin show frequent equatorial recentering of spindles in Control but not MARK2 depleted cell. Latrunculin treatment induces a mild congression defect in ~15% of cells which prolongs mitosis. OC and C refer to equatorially off-centered and centered spindles, respectively. Movies correspond to cells shown in figure 7*a*.

Supplementary Figures

Figure S1. MARK2 is enriched as dynamic membrane patches
(A) Representative still images from a time-lapse TIRF (Total Internal Reflection Fluorescence) microscopy movie of a HeLa FRT-TO cell expressing MARK2-YFP WT protein. TIRF microscopy images taken once every second show MARK2-YFP's movement as membrane patches through time (yellow arrows). Time lapse images shown (with time-frames indicated) correspond to the cell in figure 1*d* (Time-frame; 0 sec) and Supplementary Movie. Scale: uncropped image, 10 µm and cropped image, 1 µm. **(B)** Immunoblot shows the extent of MARK2 depletion following Control or MARK2 siRNA treatment as indicated and Doxycycline induced expression of MARK2-YFP in HeLa-FRT/TO cells expressing MARK2-YFP-WT (WT) or MARK2-YFP-KD (KD). Antibodies against MARK2 (upper and middle images) or gamma-tubulin (lower image) were used. Upper image alone corresponds to a reprobed blot for enhancing band intensities (*). Gamma-tubulin is used as a loading control. Percentage values in green refer to extent of total depletion of endogenous MARK2 following MARK2 siRNA treatment compared to Control siRNA treatment (normalised for protein loading using gamma-tubulin intensities). **(C)** Deconvolved Z-slice of 3D image-stacks of unperturbed interphase cells show MARK2-YFP foci at the cell-substrate interface in WT or KD mutant expressing cells, as indicated, following MARK2 siRNA treatment. Two representative cells (1 & 2) shown to highlight the striated localisation of MARK2-YFP KD mutant (blue arrow) irrespective of cell shape. Scale: uncropped image, 15 µm and cropped inset, 5 µm.

Figure S2. MARK2 KD and WT display different localisation at retraction fibres
(A) Representative Z-sections from 3D-image stacks of unperturbed mitotic cells show cell-substrate and cell cortex localisation of MARK2-YFP (WT; wild-type) or MARK2-YFP (KD; kinase dead) or MARK2-YFP (T595E) mutants, as indicated. YFP fused MARK2 protein

expression was conditionally induced in HeLa FRT/TO cell lines using Doxycycline. Widefield refers to white light images acquired to indicate the periphery of rounded up mitotic cell. Magnified image crops highlight retraction fibre length differences between MARK2 KD mutant and WT expressing cells. Scale bars: uncropped image, 15 µm and cropped image, 5µm. **(B)** Representative Z-sections from 3D-image stacks of MARK2 siRNA treated mitotic cells show cell-substrate and cell cortex localisation of MARK2-YFP (WT; wild-type) or MARK2-YFP (KD; kinase dead) as indicated. YFP fused MARK2 protein expression was conditionally induced in HeLa FRT/TO cell lines using Doxycycline. Widefield refers to white light images acquired to indicate the periphery of rounded up mitotic cell. Magnified image crops highlight retraction fibre length differences between MARK2 KD mutant and WT expressing cells. Scale: uncropped image, 15 µm and cropped image, 5µm.

Figure S3. MARK2 localisation at mitotic cortex is not dependent on LGN or cortical Dynein
(A) Experimental regime: HeLa FRT-TO MARK2-YFP cells were transfected with siRNA and induced to express MARK2-YFP using Doxycyline. Cells were treated with STLC to enrich for mitotic cells in prometaphase. **(B)** Immunoblots showing LGN protein depletion in HeLa FRT-TO cells expressing MARK2-YFP WT that were transfected with Control or LGN siRNA. **(C)** Representative live-cell images of HeLa FRT-TO cells expressing MARK2-YFP WT that were transfected with Control or LGN siRNA 72 hours prior to filming. Z-slices corresponding to mid-cortex are shown. Wide-field transmission channel and YFP channel are shown. Scale bar: 15µm

Figure S4. MARK2 re-centers pre-anaphase spindles
(A) Experimental regime: HeLa cells (His-GFP; mCherry-Tub) cells were transfected with Control or MARK2-1 siRNA oligo (as indicated) and 72 hours later imaged once every 4 minutes using a time-lapse Deconvolution microscope. Latrunculin was added 1 hour prior to imaging. Regime corresponds to data show in figure 7. **(B)** Immunoblots show the extent of MARK2 protein depletion following MARK2 siRNA treatment in HeLa cells (His-GFP; mCherry-Tub) cells treated as in **A.** Cell lysates were harvested at the end of 4-5 hour time-lapse imaging session. Blots were probed with antibodies against either MARK2 (upper image) or gamma-tubulin (lower image) as indicated. Gamma-tubulin is used as a loading control. **(C and D)** Bar graphs show proportion of cells that centered their spindles at the metaphase-anaphase transition. MARK2 siRNA treated cells center the spindles at metaphase-anaphase transition unlike Control siRNA treated cells that center their spindle pre-anaphase (B). In the presence of Lat-A, cells recenter spindles in anaphase (C). n refers to number of cells from at least three independent experimental repeats. **(E)** Empirical cumulative graph showing the proportion of equatorially Off-Centered (OC) spindles that are successfully Centered (C) within the time (in minutes) indicated. Pre-anaphase and anaphase spindle centering data are all included. Data was obtained from mitotic cells as shown in figure 7*a*. Gamma-tubulin is used as a loading control. Percentage values in green refer to extent of total depletion of endogenous MARK2 following MARK2 siRNA treatment compared to Control siRNA treatment (normalised for protein loading using gamma-tubulin intensities). **(C)** Deconvolved Z-slice of 3D image-stacks of unperturbed interphase cells show MARK2-YFP foci at the cell-substrate interface in WT or KD mutant expressing cells, as indicated, following MARK2 siRNA treatment. Two representative cells (1 & 2) shown to highlight the striated localisation of MARK2-YFP KD mutant (blue arrow) irrespective of cell shape. Scale: uncropped image, 15 µm and cropped inset, 5 µm.

Figure S1

Hart et al., 2019

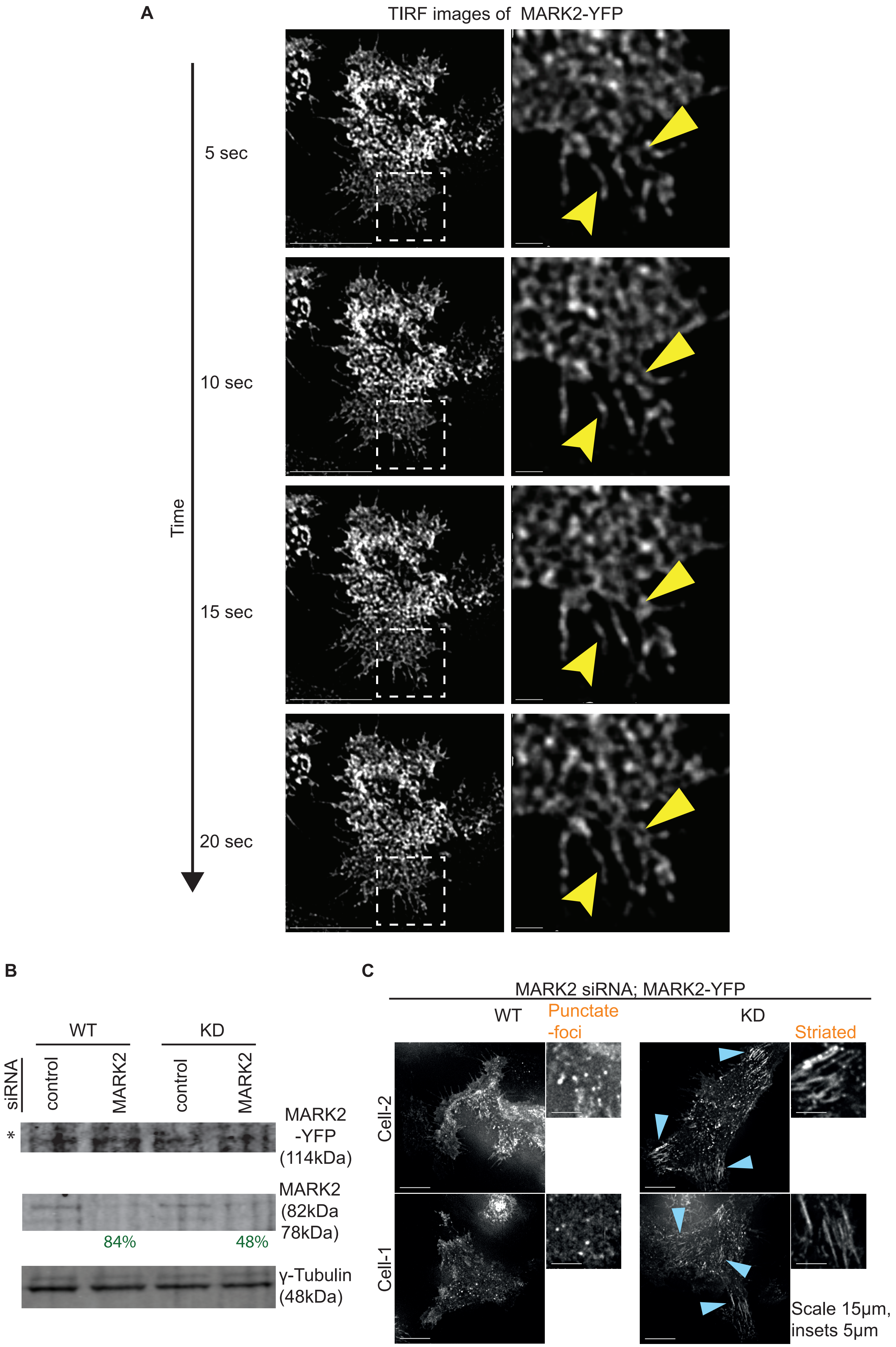

Figure S2

Hart et al., 2019

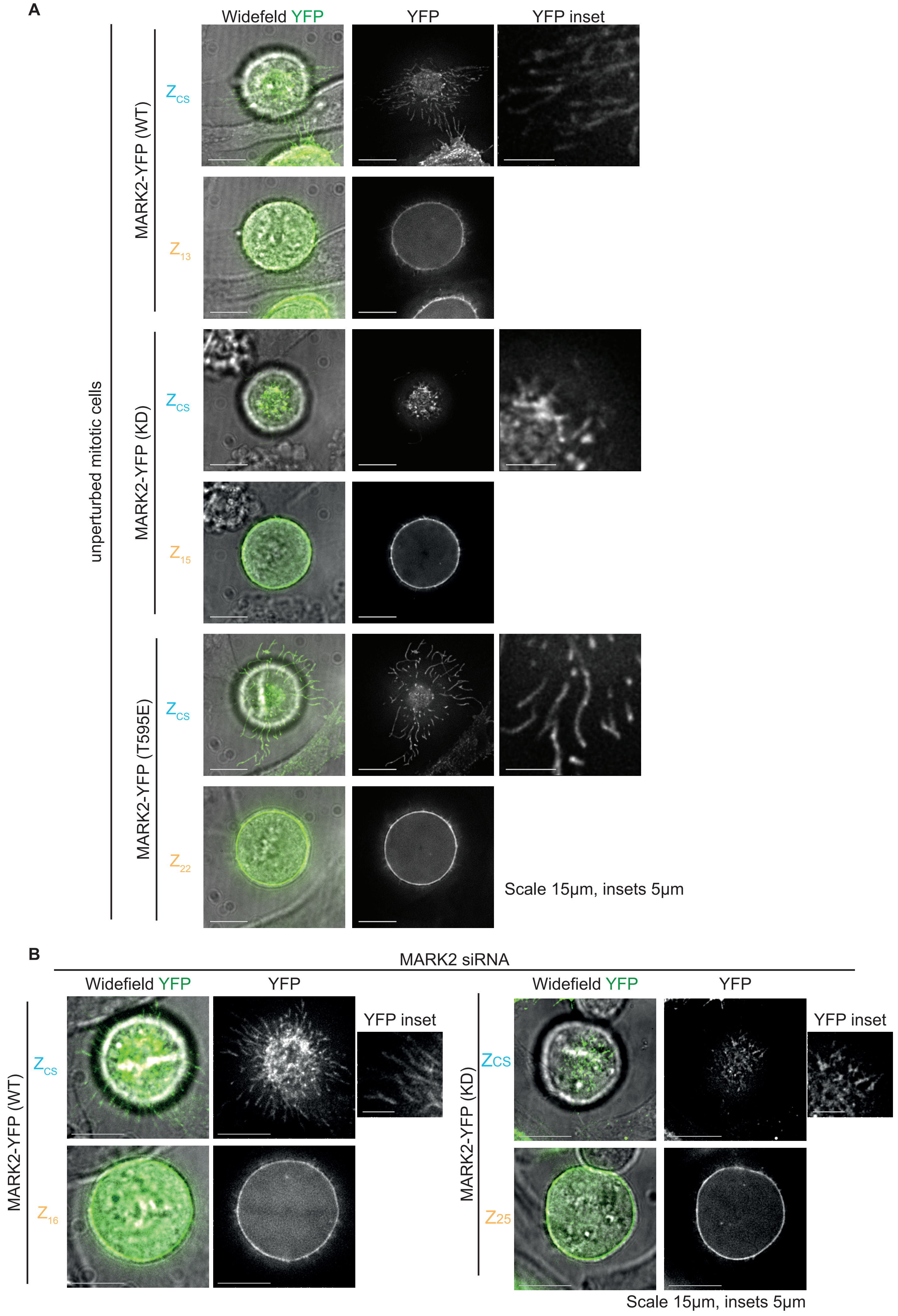

Figure S3

Hart et al., 2019

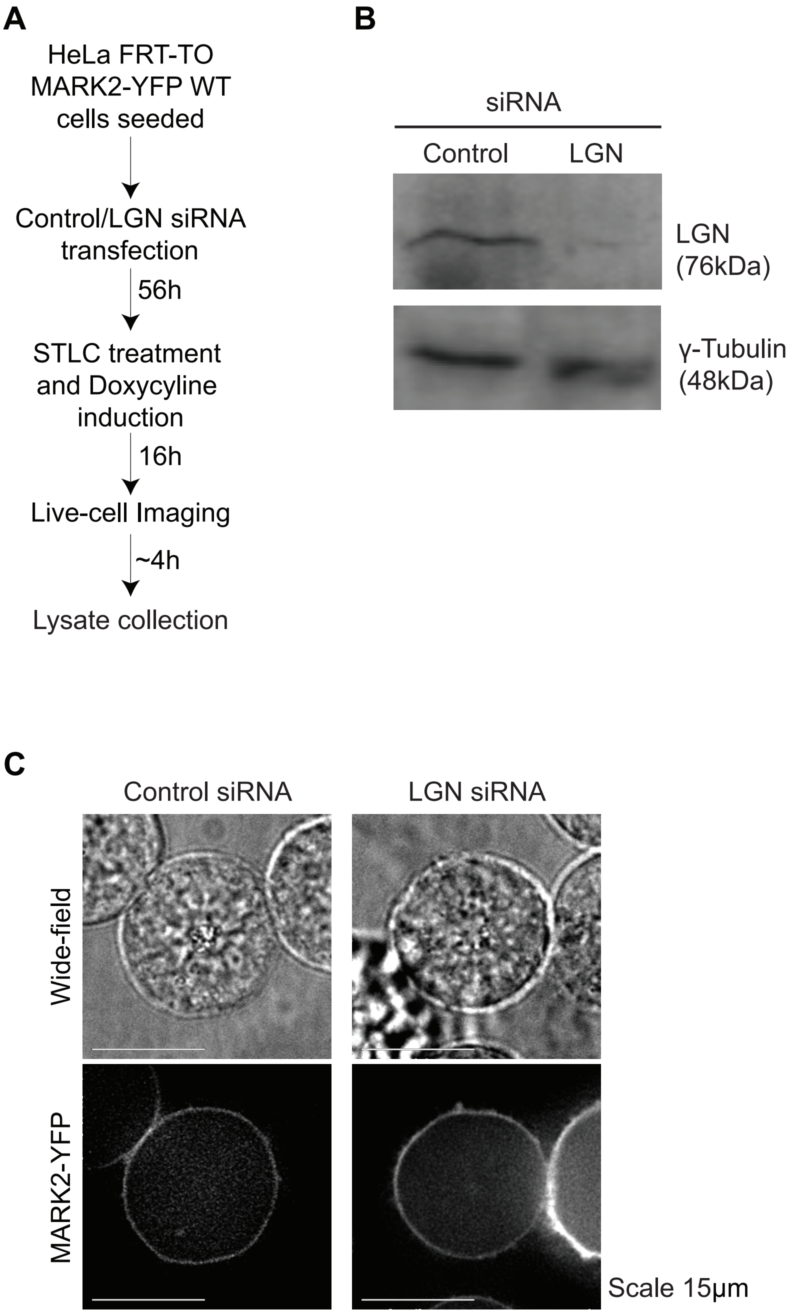

Figure S4

Hart et al., 2019

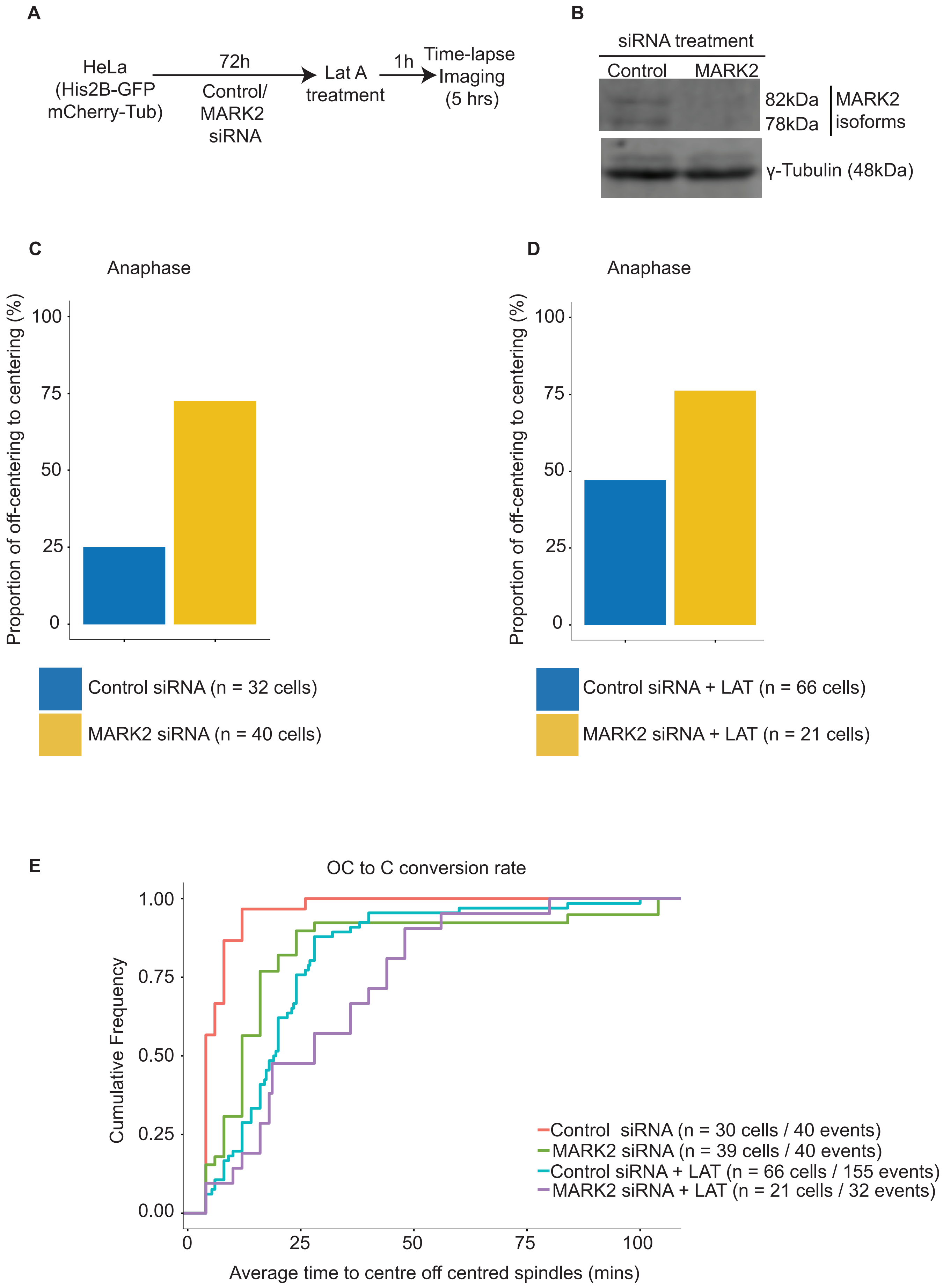

Supplement: Figures S1 - S5 [file rsob180263supp1.pdf]
